# Online respondent-driven detection for enhanced contact tracing of close-contact infectious diseases: benefits and barriers for public health practice

**DOI:** 10.1186/s12879-021-06052-4

**Published:** 2021-04-16

**Authors:** Yannick B. Helms, Nora Hamdiui, Renske Eilers, Christian Hoebe, Nicole Dukers-Muijrers, Hans van den Kerkhof, Aura Timen, Mart L. Stein

**Affiliations:** 1 National Coordination Centre for Communicable Disease Control, Centre for Infectious Disease Control, National Institute for Public Health and the Environment, Bilthoven, The Netherlands; 2 Julius Centre for Health Sciences and Primary Care, University Medical Centre Utrecht, Utrecht University, Utrecht, The Netherlands; 3 Department of Primary and Community Care, Radboud University Medical Centre, Radboud Institute for Health Sciences, Nijmegen, The Netherlands; 4 Department of Sexual Health, Infectious Diseases, and Environmental Health, South Limburg Public Health Service, Heerlen, The Netherlands; 5 Department of Social Medicine and Medical Microbiology, Care and Public Health Research Institute (CAPHRI), Maastricht University, Maastricht, The Netherlands; 6 Department of Health Promotion, Care and Public Health Research Institute (CAPHRI), Maastricht University, Maastricht, The Netherlands; 7 Athena Institute for Research on Innovation and Communication in Health and Life Sciences, VU University Amsterdam, Amsterdam, The Netherlands

**Keywords:** Contact tracing, Communicable disease control, Public health, Health professionals, Respondent-driven, eHealth, Implementation research

## Abstract

**Background:**

Online respondent-driven detection (RDD) is a novel method of case finding that can enhance contact tracing (CT). However, the advantages and challenges of RDD for CT have not yet been investigated from the perspective of public health professionals (PHPs). Therefore, it remains unclear if, and under what circumstances, PHPs are willing to apply RDD for CT.

**Methods:**

Between March and April 2019, we conducted semi-structured interviews with Dutch PHPs responsible for CT in practice. Questions were derived from the ‘diffusion of innovations’ theory. Between May and June 2019, we distributed an online questionnaire among 260 Dutch PHPs to quantify the main qualitative findings. Using different hypothetical scenarios, we assessed anticipated advantages and challenges of RDD, and PHPs’ intention to apply RDD for CT.

**Results:**

Twelve interviews were held, and 70 PHPs completed the online questionnaire. A majority of questionnaire respondents (71%) had a positive intention towards using RDD for CT. Anticipated advantages of RDD were ‘*accommodating easy and autonomous participation in CT of index cases and contact persons*’, and ‘*reaching contact persons more efficiently*’. Anticipated challenges were ‘*limited opportunities for PHPs to support, motivate, and coordinate the execution of CT*’, ‘*not being able to adequately convey measures to index cases and contact persons*’, and ‘*anticipated unrest among index cases and contact persons*’. Circumstances under which PHPs anticipated RDD applicable for CT included index cases and contact persons being reluctant to share information directly with PHPs, digitally skilled and literate persons being involved, and large scale CT. Circumstances under which PHPs anticipated RDD less applicable for CT included severe consequences of missing information or contact persons for individual or public health, involvement of complex or impactful measures for index cases and contact persons, and a disease being perceived as severe or sensitive by index cases and their contact persons.

**Conclusions:**

PHPs generally perceived RDD as a potentially beneficial method for public health practice, that may help overcome challenges present in traditional CT, and could be used during outbreaks of infectious diseases that spread via close contact. The circumstances under which CT is performed, appear to strongly influence PHPs’ intention to use RDD for CT.

**Supplementary Information:**

The online version contains supplementary material available at 10.1186/s12879-021-06052-4.

## Background

Contact tracing (CT) is a pivotal control measure in the fight against infectious diseases that transmit through contact between humans, such as measles, tuberculosis, and SARS-CoV-2 [1–3]. However, in practice CT meets certain challenges, such as the timeliness of case finding and notification, and the heavy workload associated with the CT-process for public health professionals (PHPs) [4–6].

The ongoing COVID-19 pandemic has highlighted some of these challenges. Worldwide, CT-efforts have been overwhelmed by the scale and intensity of CT required to keep up with the widespread and rapid transmission of the virus. This has greatly spurred interest in (technological) innovations that enhance CT, so that control of COVID-19, and other future outbreaks, may become more feasible.

Several novel approaches to enhance CT are currently being proposed. In particular, these involve the use of mobile CT-applications that measure and record proximity between individuals through Bluetooth, Global Positioning System-location, QR-code checkpoints, or other technologies [7]. The recorded interactions subsequently allow to (partly) automate the CT-process, for example through sending instantaneous notification messages to individuals who have been in contact with a recently confirmed COVID-19 case. Compared to ‘traditional’ CT, which relies mainly on manual labour and input from PHPs and cases, automated CT has the benefit of not being vulnerable to inaccurate and incomplete recall of contact events by cases, and reaching individuals at-risk of infection relatively quickly. However, though promising, the added value of automated CT for infectious disease control currently remains largely unproven in practice and faces challenges of its own, including its dependency on population uptake of (mobile) CT-apps, compatibility issues with older mobile phones, and various ethical, legal, and privacy concerns [7–9]. As such, the execution of CT in practice is likely to remain, at least partially, reliant on traditional CT [7].

**Fig. 1:**
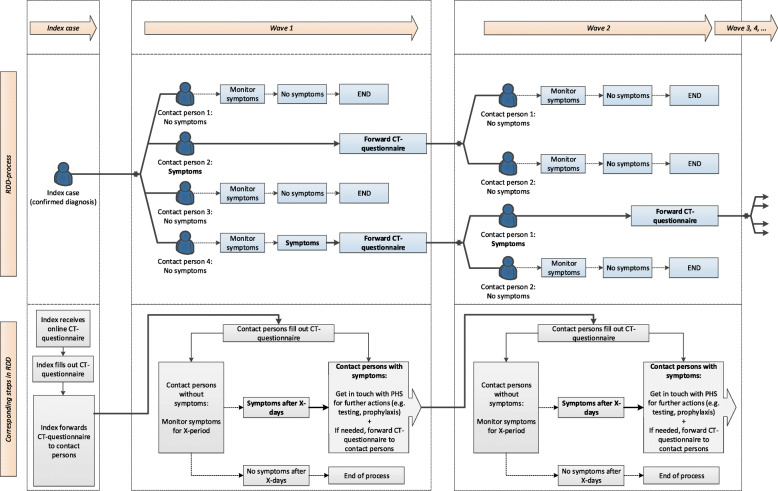
Schematic overview of RDD for CT

Online respondent-driven detection (RDD) is a novel method for case finding that may, in contrast to automated CT, enhance and support traditional CT [10, 11]. RDD was developed based on the principles of respondent-driven sampling (RDS), a snowball-type sampling method where individuals recruit ‘peers’ from their social networks. RDD starts with index cases (individuals with a confirmed infection of a given communicable pathogen), who are asked to identify and ‘recruit’ their contact persons (individuals that had contact with an index case through which transmission of a pathogen might have occurred) to fill out a web-based ‘CT-questionnaire’. The CT-questionnaire contains questions regarding disease symptoms and/or relevant behaviours (by exposure risk, e.g. visited locations), through which the risk of a potential infection may be assessed. If necessary, depending on the answers provided in the CT-questionnaire and the particular disease at hand, contact persons may be asked to 1) contact a PHP for consultation and/or testing, 2) take precautions necessary to prevent further spread of the pathogen (e.g. quarantine, implementation of hygiene routine), and 3) recruit new contact persons, and so forth. See Fig. 1 for a schematic overview of RDD for CT. With index cases and contact persons actively contributing to the execution of CT, and direct (peer-to-peer) online communications, RDD may lower the workload for PHPs and accelerate CT.

So far, RDD has been used in research settings [11–13], and its logistics for CT were tested in practice in small scale pilot studies on measles and pertussis in the Netherlands (unpublished results). However, (inter)nationally, the potential application of RDD for CT has not been systematically investigated yet from the perspective of PHPs involved in CT. As such, to understand the implementation potential of RDD for CT, a needs assessment in practice is urgently needed. Therefore, the aims of this study were to investigate 1) what advantages and challenges PHPs anticipate with regard to the use of RDD for CT in practice, 2) under what circumstances RDD may be used for CT, and 3) whether they would consider its application in practice. This study was conducted before the COVID-19 pandemic and focused on infectious diseases that spread through close contact between individuals. The key-findings of this study are summarized in Table 1.

**Table 1:** Key-findings at a glance

*How may RDD (not) benefit CT?*	PHPs anticipated that RDD may… • …accommodate easy and autonomous participation in CT by index cases and contact persons, • …increase the efficiency of CT, • …limit opportunities for PHPs to support, motivate, and coordinate the execution of CT, • …complicate conveying measures to index cases and contact persons, • …lead to unrest among index cases and contact persons.
*When may RDD be applied for CT and when not?*	• RDD may be particularly applicable in situations that involve digitally skilled and literate persons, and many contact persons. • RDD may be less applicable in situations that involve the risk of (severe) consequences for individual or public health, when complex or impactful measures may need to be taken to prevent further spread of a pathogen, and when a disease is perceived as severe or sensitive by index cases and contact persons.
*Would PHPs like to use RDD for CT in practice?*	• A majority of PHPs (71%) indicated that they would want to use RDD for CT in practice. • The circumstances under which CT is performed appear to strongly influence PHPs’ anticipated (dis)advantages of RDD and their intention to use RDD for CT in practice.

## Methods

From January to July 2019, we conducted a sequential exploratory mixed methods study [14]. First, qualitative data were collected and analysed (Phase 1). Subsequently, these data were used to develop an online questionnaire, through which we quantified our qualitative findings (Phase 2).

### Phase 1: qualitative data collection and analysis

#### Sampling

In the Netherlands, infectious disease control is executed by PHPs (nurses and doctors) working for municipal public health services (PHS). There are 25 PHS in the Netherlands, which serve different socio-geographical regions. We selected one PHS from three such regions (Utrecht, South-Holland, and Limburg) and asked them to invite two or three nurses and doctors with experience in CT for close-contact pathogens to partake in our interviews. The PHS included in this study were selected to reflect socio-demographic and -geographic diversity of PHS regions in the Netherlands, and for their willingness to participate in this study (i.e. a combination between purposive and convenience sampling was used).

#### Data collection and development of research materials

From March through April 2019, semi-structured interviews with PHPs were conducted in Dutch by one male interviewer (YBH). Interviews were held at interviewees’ respective PHS, and lasted approximately 1 h. We developed an interview guide based on the ‘innovation decision-process’ model from Rogers’ ‘diffusion of innovations’ theory [15]. Following this model, the intention to adopt RDD for CT is assumed to be influenced by 1) prior conditions, such as existing CT-practices, 2) user characteristics, such as PHPs’ age and work experience, 3) anticipated attributes (characteristics) of RDD, such as its relative advantage, compatibility, complexity, observability, and trialability, and 4) communication channels through which the innovation (RDD) is communicated to PHPs. For the purposes of this study, we focused on topics 1 through 3.

Interviews consisted of four parts. First, exploratory questions regarding PHPs’ experiences with CT were asked, focusing on their perceptions of current CT practices. Second, since at the time of the interviews most PHPs were unfamiliar with RDD, we introduced RDD to interviewees using a PowerPoint presentation designed for this purpose. The presentation consisted of a step-by-step walkthrough of the RDD process, based on Fig. 1. A standardised script for the explanation of RDD was used to ensure all interviewees received the same information (see Additional file 1). Third, the potential application of RDD for CT was discussed with interviewees in the context of three hypothetical scenarios. The use of scenarios is an effective method to elicit perceptions and attitudes towards certain actions in a real-life context [16]. To develop realistic and relatable scenarios, we developed these in collaboration with PHPs employed at the Dutch National Coordination Centre for Communicable Disease Control (LCI), which is part of the National Institute for Public Health and the Environment in the Netherlands. Based on their input, the scenarios reflected situations in CT that are relatively common in the Netherlands and are perceived to be of sufficient public health significance, in the sense that PHPs consider CT to be an appropriate intervention. In addition, in order to obtain insights into the wider applicability of RDD for CT, the scenarios differed in terms of the particular diseases at hand, with different epidemiological characteristics (e.g. transmission routes, incubation period, etc.) and respective guidelines for CT, the index case’s background (such as work and living situation), and the number and types (in terms of their risk) of contact persons potentially involved (for a detailed description of the scenarios, see Additional file 2):

- Scenario 1, ‘Scabies’: A student living in a student housing complex, who was diagnosed with scabies after having had experienced symptoms for approximately 3 months.
- Scenario 2, ‘Shigella’: A middle-aged individual who was diagnosed with shigella upon returning to his home country from an organised group holiday with friends.
- Scenario 3, ‘Mumps’: A student with a side-job as a baby-sitter, who was diagnosed with mumps.

For each scenario, interviewees were asked whether and how they would consider applying RDD for CT and why (not), and what they considered advantages and challenges of this approach. Thereafter, interviewees were asked to rank the scenarios in terms of their suitability for applying RDD, and to explain their ranking. Fourth, the potential application of RDD for CT in interviewees’ ‘day-to-day practice’ was discussed.

The interview guide and the materials used during the interviews (PowerPoint introduction to RDD and scenarios) were extensively pilot tested among a small sample of PHPs employed at the LCI.

#### Data analysis

All interviews were audio-recorded and transcribed ad verbatim. We conducted a thematic analysis to identify advantages and challenges of RDD for CT, and characteristics of scenarios that enable or restrain the use of RDD for CT [17]. Open-, axial-, and selective coding were conducted in MAXQDA 2018 v.18.0.5. No new (sub)themes emerged (i.e. data saturation was reached) after eight interviews, conducted at two PHS. Nevertheless, we conducted four more interviews, as these were all planned with the third remaining PHS. Of all interviews, 25% was randomly selected to be double coded by a second researcher (NH). Divergent findings were discussed until consensus was reached.

### Phase 2: quantitative data collection and analysis

#### Sampling

An online questionnaire was developed to quantify our main qualitative findings from Phase 1. We used an email database of the national professional network of PHPs in the Netherlands to reach respondents for the questionnaire. The database includes the vast majority of PHPs working in infectious disease control in the Netherlands, which allowed us to efficiently reach out to our target population. Between May and June 2019, 260 PHPs from all 25 PHS in the Netherlands were invited via email to complete the online questionnaire. Questionnaires were accessible to respondents for 4 weeks, during which we sent three reminders.

#### Online questionnaire

Statements were formulated regarding the qualitatively identified advantages and challenges of RDD for CT, and regarding the intention to use RDD for CT (See Additional file 3). Questionnaire respondents could respond to the statements on a 5-point Likert scale, ranging from strongly agree (1) to strongly disagree (5).

The questionnaire contained four sections. First*,* respondents were shown a webpage containing information and objectives of the study. Second*,* respondents were shown a short video explaining RDD. The video showed the same PowerPoint presentation (based on Fig. 1), and we used the same script (Additional file 1) for explaining RDD as in Phase 1. Third*,* respondents sequentially worked through the hypothetical scenarios, in each of which they responded to the developed statements. In each respective scenario, respondents were additionally asked if they would use RDD for CT if it were available at their PHS. Fourth*,* at the end of the questionnaire, we asked for respondents’ general intention (outside the context of the hypothetical scenarios) to use RDD for CT.

The online questionnaire was distributed through the survey software Formdesk (https://en.formdesk.com) and took 25–30 min to complete.

#### Statistical analyses

Descriptive analyses were conducted for respondents’ characteristics, for their responses to the statements (in each scenario), and for their intention to use RDD for CT (in each scenario and in general). Percentages were reported for all categorical variables. Distributions of continuous variables were checked using histograms, and medians and inter-quartile ranges (M;IQR) were reported (see Additional file 3).

For reporting purposes, we grouped respondents who reported to agree and respondents who reported to very much agree to the statements (on a case-by-case basis). For each statement, the combined percentage of agreeing respondents was reported. To check if the general intention to use RDD for CT was associated with respondents’ characteristics, we first created a dichotomous intention variable. Respondents who very much agreed and agreed were grouped, as were respondents who were neutral, disagreed, or very much disagreed. We then checked associations using Chi-square tests. Fisher’s exact test was used when assumptions for the Chi-square test were violated (i.e. less than 80% of categories having an expected count of five or over). All analyses were conducted in Statistical Package for the Social Sciences (SPSS) v.24.

## Results

### Study participants

Table 2 provides an overview of the sample characteristics in the qualitative and quantitative phases. We conducted twelve semi-structured interviews with PHPs; six (50%) with nurses and six (50%) with doctors. Interviewees had a median age of 38.5 years (IQR: 34.6–56.8) and a median of 9 years (IQR: 4.8–14.8) of experience with CT. Four (33.3%) interviewees were male and eight (66.7%) were female.

**Table 2:** Interviewees’ and questionnaire respondents’ characteristics

	Interviewees (*n* = 12)	Respondents (*n* = 70)
Age, in years (M;IQR)	38.5 (34.5–56.8)	49 (36–59.3)
Sex (%)		
- Male	4 (33.3)	22 (31.4)
- Female	8 (66.7)	48 (68.6)
Province of employment (%)		
- Brabant	.	6 (8.6)
- Caribbean Netherlands^a^	.	1 (1.4)
- Drenthe	.	1 (1.4)
- Flevoland	.	1 (1.4)
- Friesland	.	2 (2.9)
- Gelderland	.	14 (20.0)
- Groningen	.	5 (7.1)
- Limburg	4 (33.3)	7 (10.0)
- North-Holland	.	13 (18.6)
- Overijssel	.	6 (8.6)
- Utrecht	5 (41.7)	4 (5.7)
- Zeeland	.	1 (1.4)
- South-Holland	3 (25)	9 (12.9)
Role (%)		
- PHS nurse	6 (50.0)	33 (47.1)
- PHS doctor	6 (50.0)	35 (50.0)
- PHS manager	.	2 (2.9)
Experience with contact tracing, in years (M;IQR)	9 (4.8–14.8)	11 (6–19.3)

*M* Median, *IQR* Inter-quartile range

^a^Consisting of Sint-Eustatius, Saba, and Bonaire

We invited 260 Dutch PHS nurses and doctors to the online questionnaire. Of these, 81 started the questionnaire (response rate: 31%). Ten respondents did not complete the questionnaire and were excluded from analyses. One respondent was also excluded for giving identical answers to all questions. The final number of included respondents was 70. Respondents had a median age of 49 years (IQR: 36–59.3). Twenty-two respondents were male (31.4%) and 48 were female (68.6%). Most respondents were employed in the provinces Gelderland (20%) and North-Holland (18.6%). Thirty-three respondents (47.1%) were PHS-nurses, 35 (50%) were PHS-doctors, and two (2.9%) were department managers at a PHS. The latter two respondents were not excluded from analyses, since both reported having experience with CT. On average, respondents had a median of 11 years (IQR: 9–19.3) of experience with CT.

### Advantages and challenges of RDD for CT

Five themes related to advantages and challenges of RDD for CT were identified. Both qualitative and quantitative results are discussed in this section.

### Advantages of RDD for CT

Anticipated advantages of RDD for CT were related to: *‘accommodating easy and autonomous participation in CT for index cases and contact persons’* and *‘reaching contact persons more efficiently in CT’*. See Table 3 for illustrating quotes. See Additional file 3 for a detailed overview of quantitative results.

**Table 3:** Quotes related to advantages of RDD for CT

Themes	Illustrative quotes
Accommodating easy and autonomous participation in CT for index cases and contact persons.	“I think you can take away many barriers by having the index forward this [the online CT-questionnaire]. Especially if it is possible to do so anonymously. For example, with scabies, all the bed partners, and with mumps, all the kissing partners… We do not actually need to know all of that. They can just warn those themselves.” Nurse, mid-thirties
	“In today’s society, during the day people work, sleep, or are unavailable. This provides opportunities to go around that … so those who are hard to reach by telephone could think “this is easy, I’ll just do this tonight.” Nurse, mid-thirties
Reaching contact persons more efficiently in CT.	“I believe it’s just more efficient to handle things this way [with RDD]. And if things can be done more efficiently, that appeals to me. It saves you time.” Doctor, late-twenties
	“There is an advantage for the index. With the push of a button, he can just contact his whole group. And the information will come back quickly. So… I believe that is very efficient.” Doctor, mid-fifties

#### Accommodating easy and autonomous participation in CT for index cases and contact persons

RDD was perceived by interviewees as a method that may accommodate index case and contact person participation in CT in different ways. First, direct index-case-to-contact-person communication was perceived to lower barriers for index cases who do not want to directly share (sensitive) information about their contact persons with PHPs. Second, the use of online CT-questionnaires, especially if these may also be forwarded anonymously by index cases to their contact persons, was considered an easy and low-threshold method for informing and/or warning contact persons. Third, RDD could give index cases and contact persons the opportunity to participate in the CT-process at a moment of their choosing, rather than being dependent on contact with PHPs during office hours. Interviewees felt, however, that the aforementioned advantages may depend on the digital skills, literacy, and self-efficacy in the CT-process of index cases and contact persons. In addition, it was anticipated that if the information or warnings passed on in the CT-process would be experienced as particularly sensitive or severe by index cases or contact persons, this could inhibit their participation in CT through RDD. The latter was, for example, often considered problematic by interviewees in the mumps scenario, due to the potential involvement of at-risk infants.

A majority of questionnaire respondents believed that RDD would provide relatively easy and low-threshold options for index cases to inform and warn contact persons in all scenarios (94.3% scabies; 78.6% shigella; 52.8% mumps). In the scabies scenario, 45.6% of questionnaire respondents believed that it would be relatively pleasant for index cases and contact persons to be involved in CT through RDD, compared to 54.2% in the shigella scenario, and 32.8% in the mumps scenario.

#### Reaching contact persons more efficiently in CT

Interviewees believed that with RDD, contact persons could be reached more efficiently in CT. For example, normally PHPs are tasked with gathering contact information on, and reaching out, to contact persons (often separately). If instead index cases would self-identify their contact persons and send them a weblink to an online CT-questionnaire through ‘the push of a button’, this would save PHPs time and labour. Consequently, it was felt that with RDD an increased number of contact persons could be reached in the CT-process, in a relatively short amount of time. This was often considered particularly advantageous by interviewees in the scabies and the shigella scenarios, as they typically expected that relatively many at-risk contact persons may be involved and may need to be reached in these scenarios.

It was felt by 84.3% of questionnaire respondents that RDD could relatively save time in CT in the scabies scenario, compared to 70% in the shigella – and 50% in the mumps scenario. A lower workload through RDD was anticipated by 67.1%, 55.7%, and 37.2% of questionnaire respondents in the scabies, shigella, and mumps scenarios, respectively. In the scabies scenario, 61.4% of questionnaire respondents believed that they could reach more contact persons through RDD, compared to 55.7% in the shigella - and 42.9% in the mumps scenario.

### Challenges for CT with RDD

Anticipated challenges for CT with RDD were related to: ‘*limited opportunities for PHPs to support, motivate, and coordinate the execution of CT’, ‘not being able to adequately convey measures to index cases and contact persons’,* and ‘*anticipated unrest among index cases and contact persons’.* See Table 4 for illustrating quotes. See Additional file 3 for a detailed overview of quantitative results.

**Table 4:** Quotes related to challenges for CT with RDD

Themes	Illustrative quotes
Limited opportunities for PHPs to support, motivate, and coordinate the execution of CT.	“I do not know if you can really create a sense of urgency when you just send someone a web-link. Sometimes a PHS has a bit more authority, so that people really take it seriously.” Doctor, mid-fifties
	“You let go of the part where you yourself call someone. The part of: ‘will this be sent to the right people?, are we missing anyone?, are we not informing too many people?’ You can try to incorporate that into the system, but that danger will always remain.” Nurse, early-forties
Not being able to adequately convey measures to index cases and contact persons.	“Does someone understand what he is reading and what the consequences are? It makes you dependent of what the other person does. I do see it as an opportunity, but also as a risk to in the end not be able to execute the measures you would like to.” Nurse, early-thirties
Anticipated unrest among index cases and contact persons.	“The feeling I get of people … is that they appreciate to be talked to personally, so that we as professionals can explain why we call, and why we are asking questions. Then they can also ask their questions straight away. Then you can immediately take away a little bit of unrest. They immediately think the worst, that they are sick.” Nurse, early-forties

#### Limited opportunities for PHPs to support, motivate, and coordinate the execution of CT

Interviewees felt that CT is a complex task in which, normally, PHPs hold a central role with regard to its execution. For example, PHPs may introduce the situation at hand to index cases and their contact persons, support them where necessary, and motivate their cooperation. This, subsequently, allows PHPs to further identify and carry out CT to the right persons.

With RDD, interviewees worried that these elements of CT could potentially not be dealt with adequately. Contact between PHPs, index cases, and contact persons would be reduced, and information would mainly have to be communicated through an online CT-questionnaire. As such, index cases and contact persons would operate relatively autonomously in identifying and reaching (relevant) contact persons. Though this was not inherently considered problematic, it was felt that with limited involvement of PHPs in the CT-process, index cases and their contact persons might not take the CT-process seriously, not understand what is expected of them, or not want to cooperate. Interviewees believed that these issues could, eventually, lead to reaching irrelevant – or missing (information on) contact persons in the CT-process.

These anticipated drawbacks were perceived as particularly problematic by PHPs if the consequences of missing (information on) contact persons were potentially severe for individual or public health. For example, interviewees frequently indicated that they would personally want to be involved in the mumps scenario to oversee and coordinate the CT-process, as they were relatively concerned about the potential involvement of at-risk infants, who, in this context of mumps, were considered a high-risk group.

RDD was felt to relatively limit PHP-involvement in CT by 58.6% of questionnaire respondents in the scabies scenario, compared to 42.8% in the shigella scenario, and 64.2% in the mumps scenario. With RDD, 27.2% (scabies), 20% (shigella), and 50% (mumps) of questionnaire respondents anticipated that they could not adequately support index cases and contact persons in the CT-process. It was believed by 37.1%, 52.9%, and 32.9% of questionnaire respondents, in the scabies, shigella, and mumps scenario, respectively, that they could miss more contact persons through RDD.

#### Not being able to adequately convey measures to index cases and contact persons

With limited involvement of PHPs in the CT-process and information being communicated mainly through an online CT-questionnaire, interviewees additionally worried about adequately communicating, and subsequently delivering, recommended or required measures (e.g. instructions to seek treatment, maintain a hygiene routine, isolate/quarantine, etc.) to index cases and contact persons. If done inadequately, it was anticipated that index cases and their contact persons may, willingly or unwillingly, not (sufficiently) undertake the necessary precautions or actions. This was considered especially challenging if available measures were relatively complex or impactful. In the scabies scenario, for example, interviewees were worried about the correct implementation of simultaneous group treatment necessary to prevent re-infestation, and in the mumps scenario, interviewees worried that individuals may need a vaccination and/or need to avoid further contact with high-risk groups.

Of all questionnaire respondents, 50% (scabies), 38.6% (shigella), and 51.4% (mumps) anticipated that they could less adequately convey measures to index cases and contact persons through RDD.

#### Anticipated unrest among index cases and contact persons

Interviewees were concerned that index cases and contact persons may become overly worried when receiving a notification of potential exposure to an infectious disease, especially without introduction or support from a PHP, which could compromise the execution of CT. This was considered particularly challenging if (the consequences of) a particular disease could be experienced as severe, or sensitive (stigmatising) by index cases and contact persons. For example, interviewees often perceived a scabies infestation as potentially stigmatising for students, and in the mumps scenario, interviewees believed that the potential involvement of at-risk infants could lead to significant distress among parents and/or caretakers. In addition, concerns were expressed in regard to people excessively sharing the online CT-questionnaire, which could lead to unrest ‘spreading’ through RDD.

Of all questionnaire respondents, 74.3% (scabies), 25.7% (shigella), and 62.9% (mumps) anticipated that RDD could lead to (unnecessary) unrest among index cases and contact persons.

### Intention to use RDD for CT

Overall, interviewees indicated that, in general, they would want to use RDD for CT, as they considered it a useful, additional ‘tool’ for CT. A majority of questionnaire respondents (71%) similarly indicated that they would want to use RDD for CT in general. None of the questionnaire respondents’ characteristics were statistically significantly associated with their intention to use RDD (see Additional file 3).

Nevertheless, interviewees indicated that the potential application of RDD in practice depends on various circumstances (as outlined earlier). This was similarly reflected by questionnaire respondents, of whom 77.1% and 61.4% indicated that they would want to use RDD for CT in the scabies and the shigella scenarios, compared to 37.1% in the mumps scenario (see Additional file 3).

## Discussion

### Principal findings

This is the first study that investigates how Dutch PHPs involved in CT perceive the potential application of RDD for CT of infectious diseases that spread through close contact between individuals. RDD was anticipated to potentially benefit public health practice, as indicated by 71% of questionnaire respondents with a favourable intention towards using RDD for CT in general. PHPs anticipated that RDD could enhance CT through accommodating easy and autonomous participation in CT for index cases and their contact persons, and reaching contact persons more efficiently in CT. Challenges were anticipated in regard to limited opportunities for PHPs to support, motivate, and coordinate the execution of CT, not being able to adequately convey measures to index cases and contact persons, and anticipated unrest among index cases and contact persons. PHPs considered RDD a useful addition to CT, depending on the circumstances under which CT is applied. RDD was considered more applicable for CT when it was anticipated that index cases and contact persons are reluctant to share (sensitive) information directly with PHPs, digitally skilled and literate individuals are involved, and the scale of CT is large (many contact persons need to be reached). RDD was considered less applicable for CT when consequences of missing information or individuals in CT are potentially severe for individual or public health, when measures that index cases and contact persons need to undertake are relatively complex or impactful, and when a disease is perceived as particularly severe or sensitive by index cases and their contact persons.

RDD’s anticipated advantages and challenges for CT are, to a large extent, related to less involvement of PHPs, and conversely, a more autonomous role of index cases and contact persons in the CT-process. In a broad sense, these are commonly described topics in eHealth implementation studies [18]. However, literature specifically related to PHPs’ perceptions on the implementation of (online) innovations in CT is scarce. One closely related subject is (online) partner notification in the context of sexually transmitted diseases, which resembles RDD in the sense that index cases inform/warn their contact persons. Two studies that investigated health care providers’ perspectives of partner notification yielded similar results to those presented in this study: PHPs believed that they could reach more contact persons through partner notification on the one hand, but had concerns regarding index cases’ commitment to reach out to their contact persons on the other hand [19, 20]. These similarities indicate that strategies used to overcome challenges in the field of partner notification for sexually transmitted diseases, such as motivational interviewing with index cases prior to initiating the CT-process, may be similarly useful when applying RDD.

Based on our qualitative and quantitative results, we believe that the circumstances under which CT is performed are crucial to PHPs’ willingness to apply RDD in practice. This is illustrated by several findings. First, interviewees typically perceived the scabies and the shigella scenarios as relatively large scale, and without obvious involvement of high-risk contact persons. As such, they often felt that RDD would save more time in CT and relatively reduce PHPs workload, with limited risks for individual and/or public health. In contrast, the mumps scenario was typically perceived by interviewees as relatively small scale and potentially involved high-risk contact persons (infants), which was considered a sensitive and potentially severe situation. As such, interviewees often felt that their personal involvement would be needed to adequately support - and avoid substantial unrest among - the index case and contact persons involved. Second, in line with our qualitative findings, questionnaire respondents more frequently anticipated RDD’s benefits in the scabies and the shigella scenarios, and RDD’s disadvantages in the mumps scenario. Third, questionnaire respondents more frequently had a positive intention towards using RDD for CT in the scabies and the shigella scenarios (77.1% and 61.4% respectively), compared to the mumps scenario (37.1%). The observed differences in PHPs perception of the potential application of RDD in the different scenarios indicate that context specific research (including small scale pilot studies) and guidelines are needed for the further implementation of RDD in public health practice. Furthermore, since RDD inherently depends on participation by index cases and contact persons, we suggest additional exploration of the perspectives and needs of the general population (i.e. potential index cases and contact persons).

### Strengths and limitations

One important strength of this research was the mixed methods design, which allowed us to study qualitative findings in a larger population of PHPs [14]. We also developed and extensively pilot tested our research materials, in particular the audio-visual introduction to RDD and the hypothetical scenarios, in close collaboration with PHPs at the LCI. This allowed respondents to thoroughly deliberate on the use of RDD for CT, despite the conceptual nature of this exercise.

However, a number of limitations need to be addressed. First, at the time of this study RDD was unknown to the majority of Dutch PHPs. Therefore, it should be taken into account that the information provided to research participants through this study shaped, at least to some extent, participants’ expectations of RDD for CT in practice. We anticipated this through using a standardised script and presentation to introduce participants to RDD. In the script and presentation we only focused on the RDD process, and avoided expressions that could influence participants’ expectations of RDD’s performance in practice (e.g. we did not use phrases such as ‘RDD can improve…’). Second, this research was conducted 1 year before the COVID-19 outbreak. Considering the impact of the outbreak and the increasing interest in - and debate on technological innovations in CT, we are unsure to what degree this may have affected PHPs perceptions of RDD as presented in this study. Third, the primary goal of this study was not to quantify or disentangle which factors were the driving forces behind PHPs’ intention to use RDD for CT. An assessment of this kind would require a much larger sample than we reasonably expected to achieve. Instead, we deemed it more feasible to investigate descriptively if - and in which scenarios - PHPs would (not) want to use RDD for CT, and considered RDD’s anticipated advantages and challenges applicable. Therefore, no formal assessment (e.g. by means of a regression analysis) of the determinants of PHPs’ intention to use RDD for CT was undertaken. Fourth, the response rate to our online survey was 31%, which is comparable to healthcare professionals’ response rates to online surveys reported elsewhere [21–24]. We were unable to collect non-response data in this study. As such, we are unsure if – and to what extent non-response may have influenced our quantitative findings, for example if relatively digitally skilled and enthusiastic PHPs participated more frequently in the online questionnaire. This uncertainty should be kept in mind when interpreting our quantitative results.

### Practical considerations for further implementation of RDD for CT in practice

On a final note, we would like to stress that the implementation of RDD for CT would require sophisticated technological/IT solutions in practice. In particular, secure and privacy protecting communications between index cases, contact persons, and PHPs are (legally) required, since sensitive (medical and contact) data is digitally exchanged between these parties. In addition, the development of specific web- or mobile based ‘RDD-applications’ and functionalities may be needed. Such applications could be developed, for example, to allow PHPs to continuously develop and adapt CT-questionnaires and instruction/notification texts, to facilitate personal/anonymous notification of contact persons by index cases, or to facilitate automatic transfer of information provided by index cases and/or contact persons to case management software routinely used at a given PHS. It was, however, beyond the scope of this study to investigate these topics in meaningful detail. We, therefore, recommend researchers or PHPs interested in the application of RDD for CT in practice to explore these topics more in depth, preferably in collaboration with PHPs, experts from fields including (at least) IT and software development, user experience (UX) research, and health and privacy law.

## Conclusions

In this study, we have shown that from PHPs’ perspective, RDD may be a beneficial, additional tool for CT. Advantages of RDD for CT were anticipated in regard to accommodating easy and autonomous participation in CT for index cases and contact persons, and reaching contact persons more efficiently during CT. Challenges were anticipated in regard to limited opportunities for PHPs to support, motivate, and coordinate the execution of CT, not being able to adequately convey measures to index cases and contact persons, and anticipated unrest among index cases and contact persons. The circumstances under which CT is performed appear to strongly influence PHPs’ intention to use RDD for CT. RDD may be most beneficial when index cases and contact persons are reluctant to share (sensitive) information directly with PHPs, digitally skilled and literate individuals are involved, and the scale of CT is large (e.g. many contact persons need to be reached). RDD was considered less appropriate when consequences of missing information or individuals in CT are potentially severe for individual or public health, when measures that index cases and contact persons need to undertake are relatively complex or impactful, and when a disease is perceived as particularly severe or sensitive by index cases and their contact persons.

## Supplementary Information

**Additional file 1.** RDD explanation script and PowerPoint slides for research participants.

**Additional file 2.** Scabies, shigella & mumps hypothetical scenarios.

**Additional file 3.** Quantitative results.

## Data Availability

The quantitative dataset used and analysed during the current study is available from the corresponding author on reasonable request.

## Abbreviations

CT: Contact tracing
PHP: Public health professional
RDD: Respondent-driven detection
PHS: Public health service
LCI: Dutch National Coordination Centre for Iinfectious Disease Control
