# Supplementary material for: Online respondent-driven detection for enhanced contact tracing of close-contact infectious diseases: benefits and barriers for public health practice
Source: BMC Infect Dis. 2021 Apr 16;21:358. doi: 10.1186/s12879-021-06052-4 (PMC8051831; doi:10.1186/s12879-021-06052-4)

Additional File 1. Script and PowerPoint slides for explaining RDD to interviewees and questionnaire respondents.

Please note that the script and PowerPoint slides presented below were translated from Dutch for publication purposes.

"Respondent-driven detection (RDD) starts, like 'regular' contact tracing (CT), with an incoming notification, usually from a lab or a physician, of a patient (index) with a given communicable disease, at a public health service (PHS)."

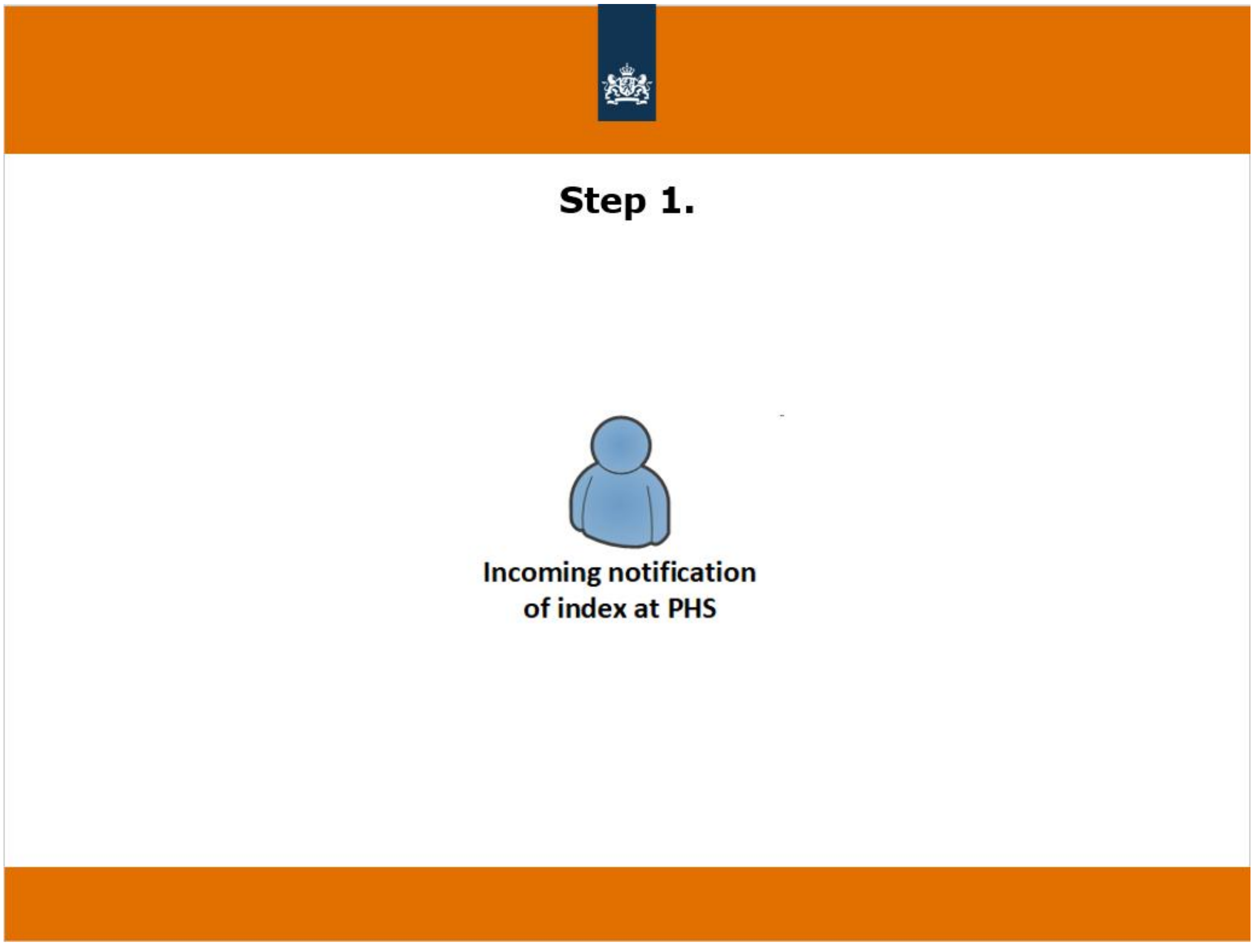

“A public health professional (PHP) then contacts the index, and sends him/her a personal link to an online questionnaire. This could, for example, be done through email, SMS, WhatsApp, etc. “

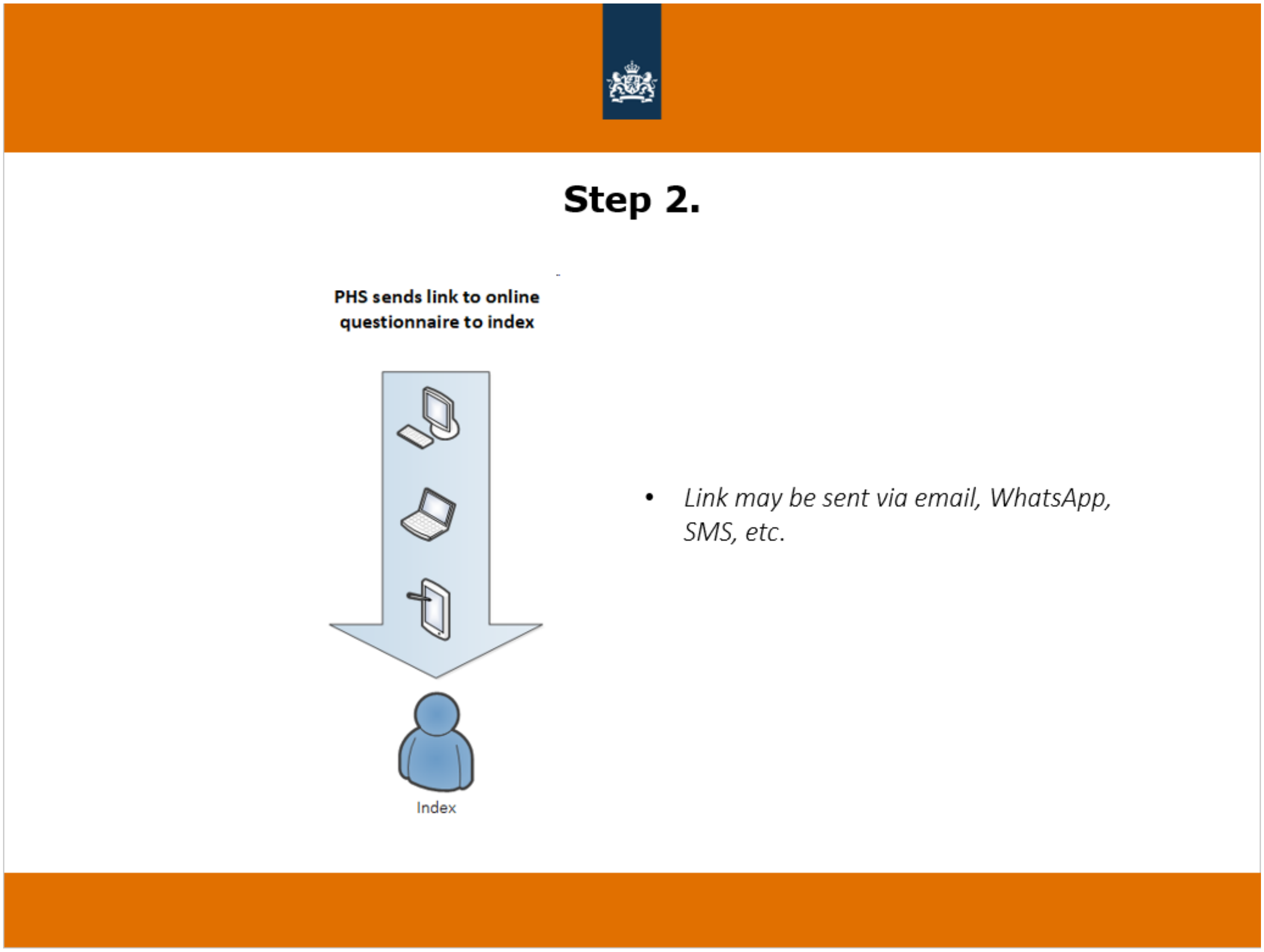

"In the online questionnaire, questions may be asked regarding specific symptoms, behaviors, and contact persons, depending on the particular disease at hand. Through the online questionnaire, the index may also be informed, for example about the disease in general, or regarding treatment and prevention options."

Step 3.

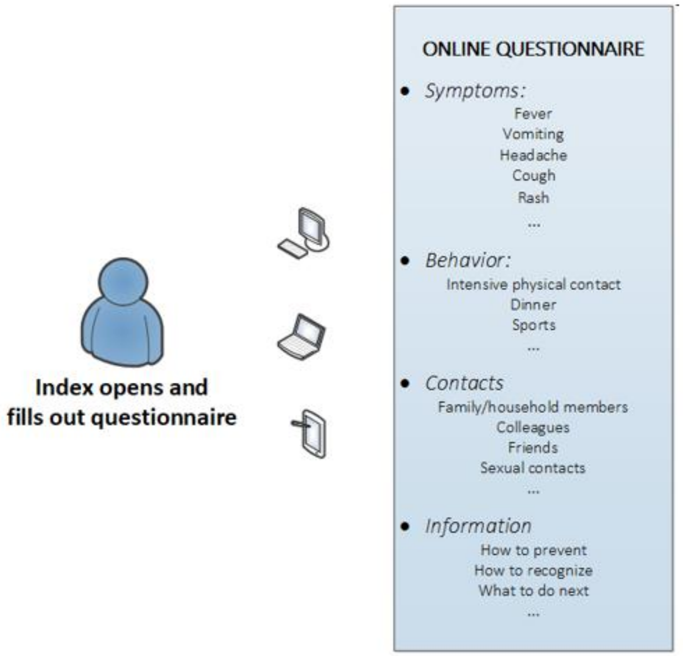

"At the end of the questionnaire, the index is asked to forward the questionnaire to relevant contact persons. This could potentially be done personally, or anonymously. If anonymous forwarding is chosen by the index, the sender could e.g. be the PHS."

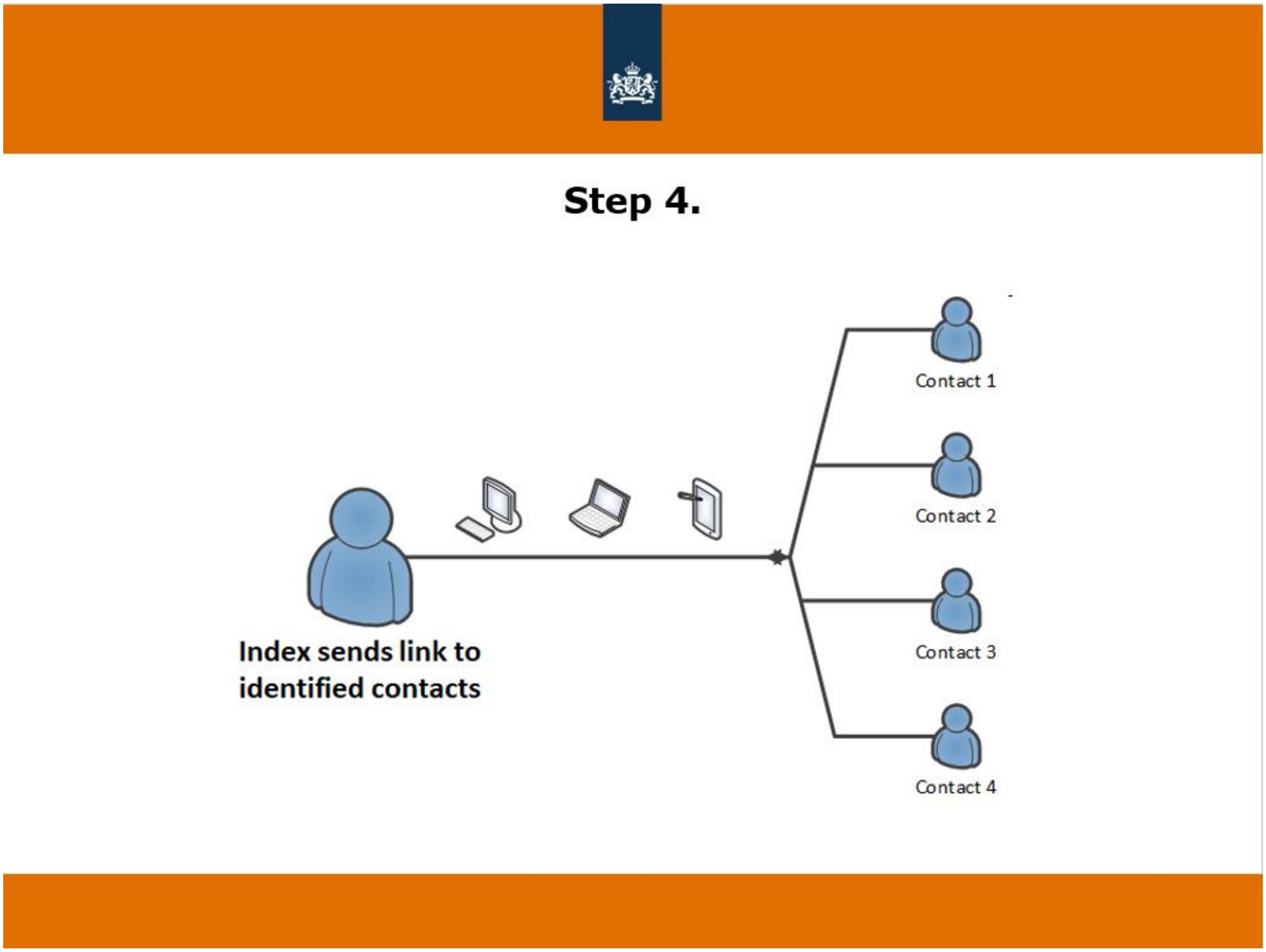

"Contact persons now fill in the online questionnaire. From here on, there are different options regarding how the RDD-process may continue, depending on what a contact person fills in."

Step 5.

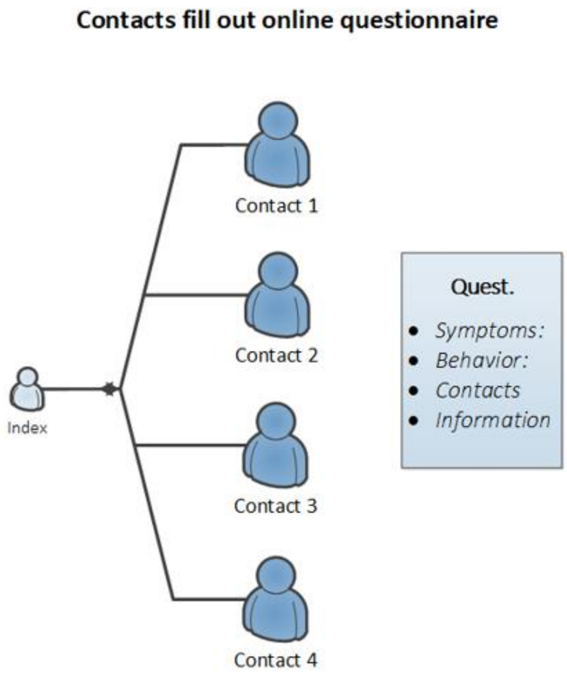

“When a contact person meets certain pre-specified criteria, for example when a certain combination of symptoms is reported, the contact person can be asked to contact a PHS, or to enter contact details, so they may be reached by a PHS. In addition, depending on answers provided in the questionnaire and after, or potentially before, lab-confirmation of the disease, the contact person may be asked to further forward the questionnaire to his/her contact persons, and so forth. When a contact person, based on answers provided, does not meet certain pre-specified requirements, for example does not have certain symptoms, he/she can be asked to fill in a follow-up questionnaire after an X-number of days. The contact person could also be asked to fill in the follow-up questionnaire when or symptoms present in the meantime, or for example, when a family member reports symptoms. If no symptoms appear within the follow-up period, the RDD process ends here. If symptoms do present, the same steps as previously explained apply.”

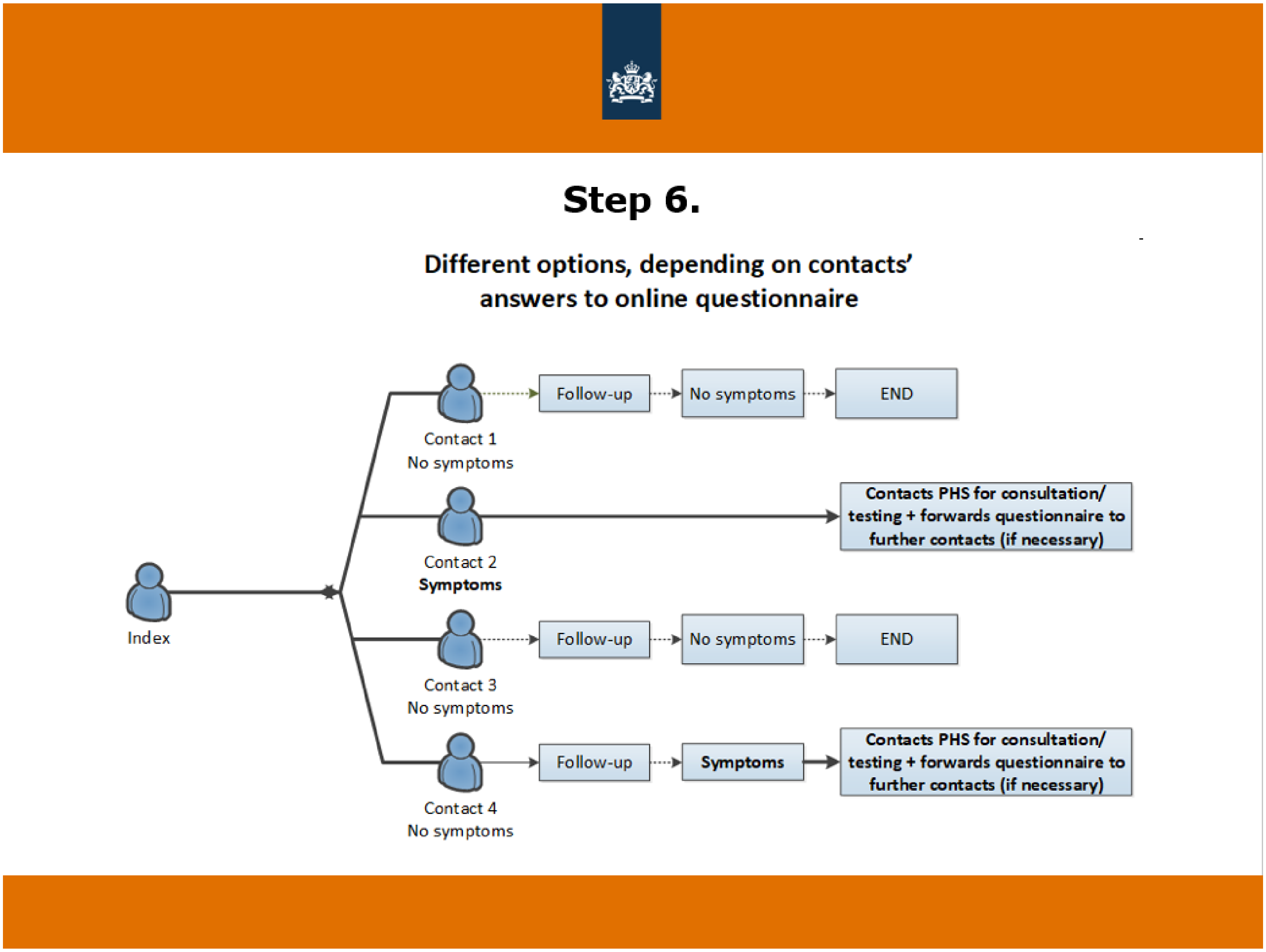

Supplement: Supplementary file 1 — Additional file 1. RDD explanation script and PowerPoint slides for research participants. [file 12879_2021_6052_MOESM1_ESM.pdf]
