# Supplementary figures and images for: Online respondent-driven detection for enhanced contact tracing of close-contact infectious diseases: benefits and barriers for public health practice
Source: BMC Infect Dis. 2021 Apr 16;21:358. doi: 10.1186/s12879-021-06052-4 (PMC8051831; doi:10.1186/s12879-021-06052-4)

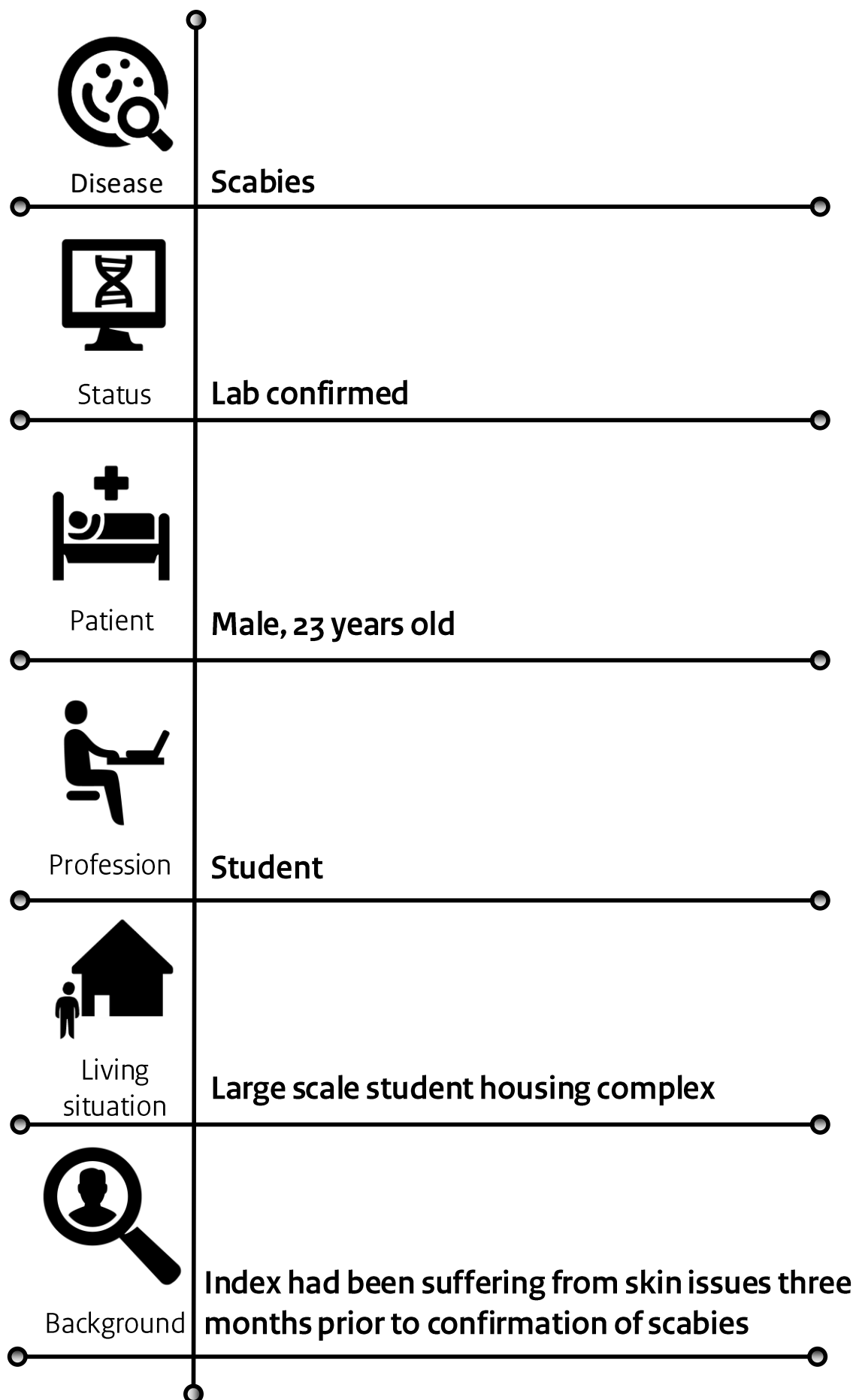

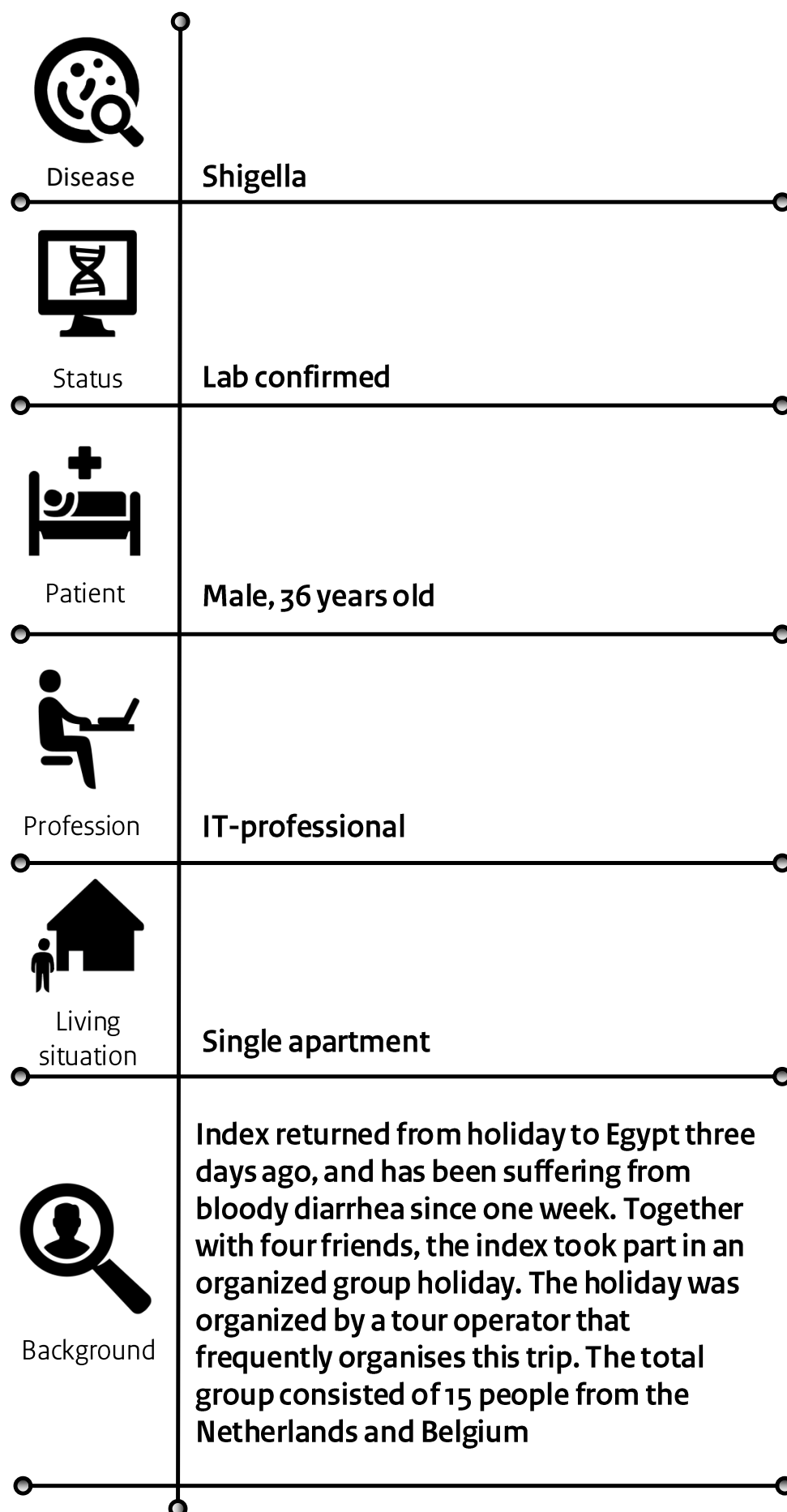

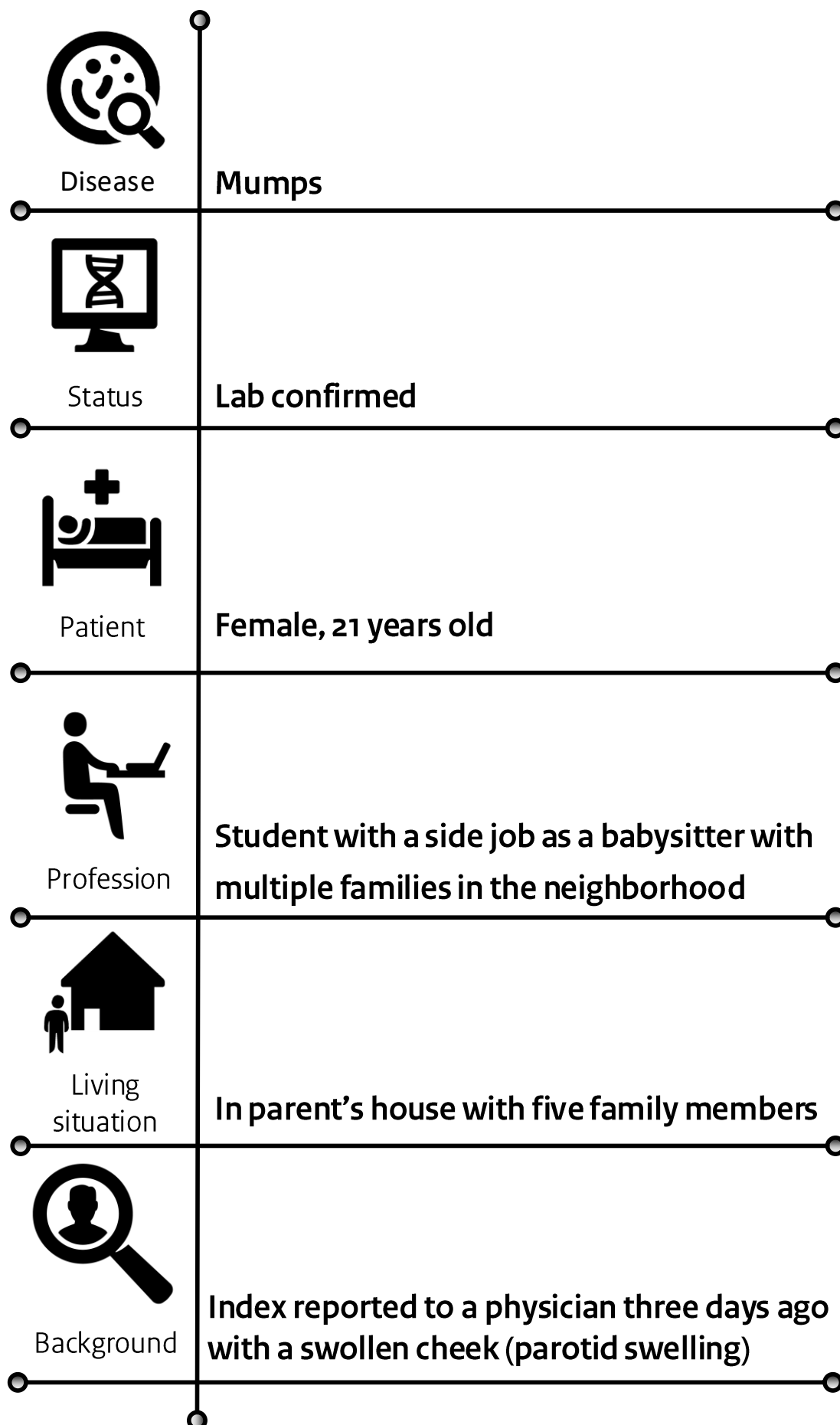

Supplement: Supplementary file 2 — Additional file 2. Scabies, shigella & mumps hypothetical scenarios. [file 12879_2021_6052_MOESM2_ESM.pdf]
