# Supplementary material for: Online respondent-driven detection for enhanced contact tracing of close-contact infectious diseases: benefits and barriers for public health practice
Source: BMC Infect Dis. 2021 Apr 16;21:358. doi: 10.1186/s12879-021-06052-4 (PMC8051831; doi:10.1186/s12879-021-06052-4)
Supplement: Supplementary file 3 — Additional file 3. Quantitative results. [file 12879_2021_6052_MOESM3_ESM.pdf]

Additional file 3, Table 1. Questionnaire respondents' responses to statements related to qualitatively identified themes.

Advantages over regular CT-methods				
Statement	*Response category*	*Response (%, n=70)*		
		SCABIES	SHIGELLA	MUMPS
'In this scenario, I think that with online-RDD I can provide low-key communication options to warn and inform patients and their contacts, compared to regular contact tracing methods.'	Very much agree<br>Agree<br>Neutral<br>Disagree<br>Very much disagree	25.7<br>68.6<br>5.7<br>0<br>0	18.6<br>60<br>12.9<br>7.1<br>1.4	11.4<br>41.4<br>17.1<br>30<br>0
'In this scenario, I think that with online-RDD I can involve patients and their contacts in contact tracing in a way more pleasant to them way, compared to regular contact tracing methods.'	Very much agree<br>Agree<br>Neutral<br>Disagree<br>Very much disagree	4.3<br>41.3<br>40<br>12.9<br>1.4	7.1<br>47.1<br>28.6<br>15.7<br>1.4	5.7<br>27.1<br>30<br>32.9<br>4.3
'In this scenario, I think that I can save time with online-RDD, compared to regular contact tracing methods.'	Very much agree<br>Agree<br>Neutral<br>Disagree<br>Very much disagree	17.1<br>65.7<br>12.9<br>2.9<br>1.4	15.7<br>54.3<br>18.6<br>11.4<br>0	14.3<br>35.7<br>22.9<br>21.4<br>5.7
'In this scenario, I think that with online-RDD I can reach more contacts of patients, compared to regular contact tracing methods.'	Very much agree<br>Agree<br>Neutral<br>Disagree<br>Very much disagree	14.3<br>47.1<br>28.6<br>10<br>0	14.3<br>41.4<br>28.6<br>15.7<br>0	12.9<br>30<br>15.7<br>41.3<br>0
'In this scenario, I think that online-RDD can reduce the workload for public health professionals (PHPs), compared to regular contact tracing methods.'	Very much agree<br>Agree<br>Neutral<br>Disagree<br>Very much disagree	7.1<br>60<br>27.1<br>4.3<br>1.4	10<br>45.7<br>27.1<br>15.7<br>1.4	2.9<br>34.3<br>34.3<br>25.7<br>2.9
Challenges for CT with online-RDD				
'In this scenario, I think that using online-RDD can go at the expense of my contact with patients and their contacts, compared to regular contact tracing methods.'	Very much agree<br>Agree<br>Neutral<br>Disagree<br>Very much disagree	2.9<br>55.7<br>30<br>10<br>1.4	1.4<br>41.4<br>35.7<br>20<br>1.4	7.1<br>57.1<br>30<br>4.3<br>1.4
'In this scenario, I think that with online-RDD I cannot offer adequate support to patients and their contacts, compared to regular contact tracing methods.'	Very much agree<br>Agree<br>Neutral<br>Disagree<br>Very much disagree	2.9<br>24.3<br>40<br>32.9<br>0	2.9<br>17.1<br>40<br>38.6<br>1.4	4.3<br>45.7<br>25.7<br>21.4<br>2.9
'In this scenario, I think that with online-RDD I can miss important contacts, compared to regular contact tracing methods.'	Very much agree<br>Agree<br>Neutral<br>Disagree<br>Very much disagree	1.4<br>35.7<br>37.1<br>25.7<br>0	14.3<br>38.6<br>42.9<br>4.3<br>0	2.9<br>30<br>30<br>34.3<br>2.9
'In this scenario, I think that with online-RDD I can convey measures to patients and their contacts less adequately, compared to regular contact tracing methods.'	Very much agree<br>Agree<br>Neutral<br>Disagree<br>Very much disagree	10<br>40<br>28.6<br>21.4<br>0	4.3<br>34.3<br>32.9<br>27.1<br>1.43	7.1<br>44.3<br>25.7<br>21.4<br>1.4
'In this scenario, I think that online-RDD can lead to unnecessary unrest with patients and their contacts, compared to regular contact tracing methods.'	Very much agree<br>Agree<br>Neutral<br>Disagree<br>Very much disagree	0<br>34.3<br>40<br>24.3<br>1.4	1.4<br>24.3<br>41.4<br>28.6<br>4.3	11.4<br>51.4<br>24.3<br>11.4<br>1.4
Intention				
'In this scenario, I would like to make use of online-RDD for CT if it were available at my PHS'	Very much agree<br>Agree<br>Neutral<br>Disagree<br>Very much disagree	10<br>67.1<br>18.6<br>2.9<br>1.4	12.9<br>48.6<br>30<br>7.1<br>1.4	15.7<br>21.4<br>27.1<br>32.9<br>2.9

Additional file 3, Table 2. Bivariate associations between questionnaire respondents' characteristics and intention to use online-RDD for CT.

	Positive intention (n=50)	Neutral/Negative intention (n=20)	Test statistic	Df	P-value
**Age group** (%)			Exact= 7.32	-	0.059
- 25-34 years	12 (24)	1 (5)			
- 35-44 years	13 (26)	2 (10)			
- 45-54 years	10 (20)	6 (30)			
- ≥ 55 years	15 (30)	11(55)			
**Sex** (%)			X^2 =0.54	1	0.46
- Male	17 (34)	5 (25)			
- Female	33 (66)	15 (75)			
**Province of employment** (%)			Exact= 16.45	-	0.09
- Brabant	6 (12)	.			
- Caribbean Netherlands*	1 (2)	.			
- Drenthe	.	1 (5)			
- Flevoland	1 (2)	.			
- Friesland	1 (2)	1 (5)			
- Gelderland	10 (20)	4 (20)			
- Groningen	1 (2)	4 (20)			
- Limburg	5 (10)	2 (10)			
- North-Holland	9 (18)	4 (20)			
- Overijssel	6 (12)	.			
- Utrecht	3 (6)	1 (5)			
- Zeeland	.	1 (5)			
- South-Holland	7 (14)	2 (10)			
**Role** (%)			X^2 =1.1	1	0.3
- Nurse	23 (51.1)	7 (36.8)			
- Doctor	22 (48.9)	12 (63.2)			
**Experience with CT** (%)			Exact= 6.97	-	0.13
- 0-5 years	13 (26)	3 (15)			
- 6-10 years	14 (28)	4 (20)			
- 11-15 years	12 (24)	2 (10)			
- 16-20 years	5 (10)	5 (25)			
- ≥ 21 years	6 (12)	6 (30)			
